# Selective Activation of M_1_ Muscarinic Receptors Attenuates Human Colon Cancer Cell Proliferation

**DOI:** 10.3390/cancers15194766

**Published:** 2023-09-28

**Authors:** Margaret H. Sundel, Natalia Sampaio Moura, Kunrong Cheng, Oscar Chatain, Shien Hu, Cinthia B. Drachenberg, Guofeng Xie, Jean-Pierre Raufman

**Affiliations:** 1 Department of Surgery, University of Maryland School of Medicine, Baltimore, MD 21201, USA; maggie.sundel@som.umaryland.edu; 2 Department of Medicine, Division of Gastroenterology and Hepatology, University of Maryland School of Medicine, Baltimore, MD 21201, USA; natalia.sampaiomoura@som.umaryland.edu (N.S.M.); kcheng@som.umaryland.edu (K.C.); olchatain@liberty.edu (O.C.); hushien@gmail.com (S.H.); gxie@som.umaryland.edu (G.X.); 3 Department of Pathology, University of Maryland School of Medicine, Baltimore, MD 21201, USA; cdrachenberg@som.umaryland.edu; 4 VA Maryland Healthcare System, Baltimore, MD 21201, USA; 5 Marlene and Stewart Greenebaum Cancer Center, University of Maryland School of Medicine, Baltimore, MD 21201, USA; 6 Department of Biochemistry and Molecular Biology, University of Maryland School of Medicine, Baltimore, MD 21201, USA

**Keywords:** colon cancer, muscarinic receptors, cell proliferation, carcinogenesis

## Abstract

**Simple Summary:**

M_1_ and M_3_ muscarinic receptors, M_1_R and M_3_R, play important roles in health and disease. Previously, we found opposite patterns of expression in colon cancer for the genes encoding M_1_R and M_3_R. Likewise, M_1_R and M_3_R deficiency had opposite effects in a mouse colon cancer model. Based on these observations, we hypothesized that activating M_1_R might inhibit the growth of colon cancer cells. Here, we confirmed divergent expression of M_1_R and M_3_R protein in progressive colon cancer. Then, we discovered that in contrast to M_3_R activators, treating human colon cancer cells with M_1_R activators inhibited cell growth. The effects of M_1_R activation appeared to be mediated by a signaling pathway downstream of the receptor. Notably, M_1_R activation was more effective than conventional chemotherapy agents at inhibiting colon cancer cell growth and combining these agents augmented this action. We believe our findings support further investigation of selective M_1_R activators as treatments for advanced colon cancer.

**Abstract:**

M_3_ muscarinic receptor (M_3_R) activation stimulates colon cancer cell proliferation, migration, and invasion; M_3_R expression is augmented in colon cancer and ablating M_3_R expression in mice attenuates colon neoplasia. Several lines of investigation suggest that in contrast to these pro-neoplastic effects of M_3_R, M_1_R plays an opposite role, protecting colon epithelial cells against neoplastic transformation. To pursue these intriguing findings, we examined the relative expression of M_1_R versus M_3_R in progressive stages of colon neoplasia and the effect of treating colon cancer cells with selective M_1_R agonists. We detected divergent expression of M_1_R and M_3_R in progressive colon neoplasia, from aberrant crypt foci to adenomas, primary colon cancers, and colon cancer metastases. Treating three human colon cancer cell lines with two selective M_1_R agonists, we found that in contrast to the effects of M_3_R activation, selective activation of M_1_R reversibly inhibited cell proliferation. Moreover, these effects were diminished by pre-incubating cells with a selective M_1_R inhibitor. Mechanistic insights were gained using selective chemical inhibitors of post-muscarinic receptor signaling molecules and immunoblotting to demonstrate M_1_R-dependent changes in the activation (phosphorylation) of key downstream kinases, EGFR, ERK1/2, and p38 MAPK. We did not detect a role for drug toxicity, cellular senescence, or apoptosis in mediating M_1_R agonist-induced attenuated cell proliferation. Lastly, adding M_1_R-selective agonists to colon cancer cells augmented the anti-proliferative effects of conventional chemotherapeutic agents. Collectively, these results suggest that selective M_1_R agonism for advanced colon cancer, alone or in combination with conventional chemotherapy, is a therapeutic strategy worth exploring.

## 1. Introduction

Members of the cholinergic muscarinic receptor family, expressed ubiquitously in all organs and tissues examined, play key roles in regulating important normal and neoplastic cell functions [1]. Of the five cholinergic muscarinic receptor subtypes (designated M_1_R–M_5_R and encoded respectively by *CHRM1*–*CHRM5*), M_1_R, M_3_R, and M_5_R, often referred to as MR_odd_, are coupled to G_q_ and downstream signaling is mediated by phospholipid turnover which increases cellular calcium levels that, in turn, activates a variety of protein kinases. In contrast, M_2_R and M_4_R (MR_even_), coupled to G_s_, modulate protein kinase activity by altering cellular levels of cAMP. Notably, few studies have examined differences between the actions of muscarinic receptor subtypes within these two subfamilies.

Amongst other key functions in normal gastrointestinal biology, muscarinic receptors regulate cell proliferation, migration, secretion, absorption, and intestinal stem cell differentiation [2]. In gastrointestinal neoplasia, muscarinic receptors regulate cell proliferation, survival, migration, and invasion [3]; the latter being hallmarks of an invasive phenotype. In human colon cancer cell lines, signaling via M_3_R selectively stimulates the expression and release of matrix metalloproteinase (MMP)1, MMP7, and other proteases that facilitate colon cancer cell migration and invasion. Treating human colon cancer cells with non-subtype-selective muscarinic agonists (e.g., acetylcholine or bethanechol) stimulates cell proliferation, invasion, and intestinal tumor formation in murine colon cancer models. M_3_R overexpression by primary colon adenocarcinomas predicts metastatic disease and M_3_R-deficient mice and mice treated with a non-selective muscarinic receptor inhibitor (scopolamine) exhibit attenuated intestinal neoplasia [3].

In contrast to M_3_R, much less is known about the functional role of M_1_R expression and activation in health and disease. Previously, it was considered that the functions of M_1_R and M_3_R overlapped, i.e., that their activation resulted in similar downstream effects with variable efficacy, and that this redundancy presumably provided survival advantages to the organism. For example, work using mice with global knockout of M_1_R, M_3_R, and combined deficiency of both M_1_R and M_3_R revealed that genetic ablation of both receptor subtypes was required to completely attenuate cholinergic agonist-induced pepsinogen secretion. These findings suggested overlapping activities of these two muscarinic receptor subtypes.

The current line of investigation was instigated by our surprising finding that in contrast to the pro-neoplastic effects of M_3_R, M_1_R expression appeared to play an opposite role, protecting colon epithelial cells against neoplasia. In an unbiased analysis, we found that in contrast to *CHRM3*, expression of *CHRM1* was significantly downregulated in human colorectal adenocarcinomas [4]. In line with these observations, using a murine colon cancer model, we had previously reported that global M_1_R deficiency resulted in a modest increase in the number of colon tumors, and in mice with dual M_1_R and M_3_R deficiency, global M_1_R deficiency negated the anti-neoplastic effects of M_3_R knockout; that is, in contrast to azoxymethane (AOM)-treated M_3_R-deficient mice that had fewer colon tumors than AOM-treated wild-type (WT) control mice, AOM-treated mice with dual deficiency of M_1_R and M_3_R had as many colon tumors as control mice [5]—that is, M_1_R deficiency reversed the anti-neoplastic effects of M_3_R deficiency. Thus, in contrast to work from several laboratories supporting the concept that M_3_R is an oncogene and tumor promoter [6,7,8], our findings suggest that within the context of colorectal cancer, M_1_R functions as a tumor suppressor. This intriguing paradigm is supported by our discovery of additional instances where M_1_R and M_3_R have diametrically divergent effects; for example, whereas activation of M_3_R in murine models of advanced liver disease promotes fibrosis and advanced disease, M_1_R activation mitigates liver scarring.

As we report in the present communication, to pursue these interesting observations, we examined the relative expression of M_1_R versus M_3_R in progressive stages of human colon neoplasia. We then examined the actions of activating M_1_R with selective receptor agonists. When relevant, we compared results to those obtained using non-subtype-selective muscarinic receptor agonists and antagonists. The overarching goal was to elucidate the relative contribution of M_1_R expression and activation to colon cancer cell function. This work uncovered divergent expression of M_1_R and M_3_R in progressive colon neoplasia, from aberrant crypt foci to adenomas, primary colon cancers, and colon cancer metastases. Then, using well-studied, established colon cancer cell lines we found that in contrast to M_3_R, selective activation of M_1_R inhibited human colon cancer cell proliferation. Confirmatory evidence was obtained by showing similar effects when applying two selective M_1_R agonists to three different colon cancer cell lines, and attenuation of these effects when we pre-incubated cells with a selective inhibitor of M_1_R activation. We gained mechanistic insights using selective chemical inhibitors of post-receptor signaling pathways and assessing M_1_R-dependent changes in the activation (phosphorylation) of key downstream kinases. We also compared the actions of M_1_R-selective agonists alone and in combination with conventional chemotherapeutics for colon cancer, 5-fluorouracil and oxaliplatin. Our results suggest that the selective M_1_R agonism may be a worthwhile treatment strategy for advanced colon cancer that is worthy of further exploration.

## 2. Materials and Methods

### 2.1. Reagents and Antibodies

Antibodies against total and phospho-p38 MAPK (catalog #9212 and 9211), total and phospho-ERK1/2 (catalog #4695 and 4377), and β-actin (catalog #93473) were purchased from Cell Signaling (Danvers, MA, USA). Acetylcholine (ACh), atropine, McN-A-343 (McN), xanomeline, VU0255035 (VU), Gö6976, SB203580, and unspecified reagents were purchased from Sigma-Aldrich (Burlington, MA, USA).

### 2.2. Cell Lines and Cell Culture

HT-29, H508, and HCT116 human colon cancer cell lines were purchased from American Type Culture Collection (ATCC) (Manassas, VA, USA). H508 cells were grown in RPMI 1640 (Thermo Fisher Scientific (Waltham, MA USA)) supplemented with 10% fetal bovine serum (FBS). HT-29 cells were grown in McCoy’s 5A medium (Thermo Fisher Scientific) supplemented with 10% FBS. HCT116 cells were grown in McCoy’s 5A medium (Thermo Fisher Scientific) supplemented with 10% FBS plus 50 µg/mL streptomycin and 50 U/mL penicillin. Cells were grown at 37 °C, with 5% CO_2_ in a humidified incubator and passaged weekly at subconfluence after trypsinization. Prior to each experiment, the cells were incubated in serum-free medium without FBS for 24 h for serum starvation.

### 2.3. Human Tissues

To examine M_1_R (*CHRM1*) and M_3_R (*CHRM3*) gene and protein expression, we used archived pre-existing de-identified surgical specimens of colon cancer and adjacent normal colon epithelium (approved by the University of Maryland School of Medicine Institutional Review Board and the Baltimore VA Research and Development Committee). To ensure we had sufficient material for analysis and uniform distribution of immunohistochemical staining, we used only surgical tissue specimens; we did not use smaller, endoscopic biopsies.

### 2.4. Immunoblot Analysis

After treatments with muscarinic receptor agonists, cells were lysed in a solution containing Cell Lysis Buffer (catalog #9803), anti-protease (catalog #5871), and anti-phosphatase (catalog #5870)—all from Cell Signaling. Cell lysates were centrifuged at 15,000× *g* at 4 °C for 10 min. Supernatants were collected and protein concentration was determined by the BCA method (Thermo Fisher Scientific). Proteins were separated by SDS-PAGE and transferred to PVDF membranes that were probed against total and phospho-38 MAPK, total and phospho-ERK1/2, and total and cleaved caspase-3. To confirm equal protein loading, blots were stripped and re-probed with antibodies against β-actin. Immunoblots were then developed using the Bio-Rad ChemiDoc Touch Imaging System (Bio-Rad Laboratories, Hercules, CA, USA). In most experiments, conditions were normalized and expressed relative to the positive control.

### 2.5. Quantitative Real-Time PCR (qPCR)

qPCR was performed on total RNA obtained from H508, HT-29, and HCT116 human colon cancer cells. We synthesized first-strand cDNAs from 5 µg RNA (Superscript III First Strand Synthesis System for RT-PCR, Invitrogen (Carlsbad, CA, USA)) and performed qPCR using 50 ng cDNA, the SYBR Green PCR Master Mix (Applied Biosystems (Foster City, CA, USA)), and 0.5 μM in 20 μL forward and reverse primers. We designed primers (Supplementary Table S1) to span introns using the National Center for Biotechnology Information nucleotide database SIM-4 gene alignment program and on-line software (www.genscript.com/ssl-bin/app/primer (accessed on 6 September 2012). We performed qPCR using Step One (Applied Biosystems) with Power SYBR Green Master Mix (ABI) and the following PCR conditions: 5 min at 95 °C followed by 37 cycles of 95 °C for 15 s, 60 °C for 20 s, 72 °C for 40 s, and final cycles at 95 °C for 15 s, 60 °C for 15 s, and 95 °C for 15 s. We normalized gene expression to *β*_2_-*microglobulin* (*B2M*) and analyzed qPCR data using the comparative C_T_ (2^−ΔΔCT^) method.

### 2.6. Cell Proliferation Assay

Cell proliferation was measured using the WST-1 Cell Proliferation Assay Kit (Roche (Atlanta, GA, USA)), following the manufacturer’s instructions. H508, HT-29, and HCT116 cells were cultured at 50% confluence on 96-well flat-bottom plates. After serum starvation overnight, cells were treated with test chemicals for five days with or without 30-min pretreatment with inhibitors. Plates were read on a microplate reader at 440 nm 30 min after adding the WST-1 reagent. Cell proliferation rates were calculated according to the manufacturer’s protocol.

### 2.7. Lactate Dehydrogenase (LDH) Release Assay

Cytotoxicity was measured using the CyQUANT LDH Cytotoxicity Assay (Invitrogen). H508 cells were cultured at 50% confluence on 96-well flat-bottom plates. After serum starvation overnight, cells were treated with test chemicals for five days. The assay was performed per the manufacturer’s instructions; briefly, 50 µL of sample medium was removed from each well and treated with the reaction mixture followed by a 30-min incubation at room temperature. After 30 min, stop solution was added and the plates were examined using a microplate reader set at 490 and 680 nm. Percent cytotoxicity was determined using the manufacturer’s instructions.

### 2.8. Senescence-Associated β-Galactosidase

Cell senescence was evaluated using the Senescence β-Galactosidase Staining Kit (Cell Signaling). H508 cells were cultured at 50% confluence in six-well plates. After serum starvation overnight, cells were treated with test agents for 24, 48, and 72 h. Cells were fixed and stained following the manufacturer’s instructions and images were captured using a Nikon Eclipse Ti microscope (Melville, NY, USA).

### 2.9. Statistical Analysis

Data are presented as mean ± SE of at least three separate experiments. As appropriate for comparisons and specified in figure legends, data were analyzed using two-tailed unpaired Student’s *t*-test. We used global nonlinear regression to compare dose response curves. These analyses were performed using GraphPad Prism version 9.5.1 for macOS, GraphPad, Boston, MA, USA. We considered *p* < 0.05 to be statistically significant.

## 3. Results

### 3.1. Relative CHRM1 and CHRM3 mRNA Expression in Colorectal Adenocarcinomas

**Figure 1:**
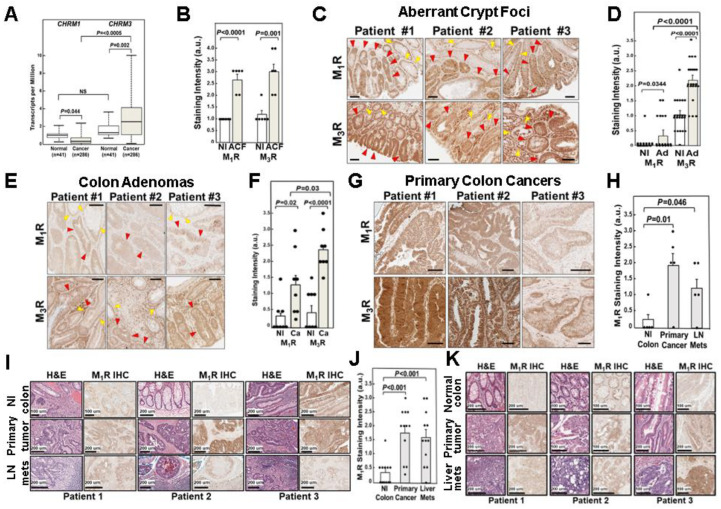
Divergent *CHRM1* and *CHRM3* mRNA and M_1_R and M_3_R protein expression in progressive colon neoplasia compared to a normal colon. (**A**) Reduced expression of the gene encoding M_1_R (*CHRM1*) and overexpression of the gene encoding M_3_R (*CHRM3*) in colorectal cancer. As shown by box-whisker plots, UALCAN server analysis [9] reveals *CHRM1* (**left**) and *CHRM3* (**right** panel) mRNA transcripts are respectively under- and over-expressed in primary colon cancer (n = 286 patients) compared with a normal colon (n = 41 patients). For a normal colon, maximum, upper quartile, median, lower quartile, and minimum values, respectively, are 2.146, 1.375, 1.03, 0.828, and 0.299 for *CHRM1* and 3.645, 2.055, 1.272, 1.069, and 0.678 for *CHRM3*. For colon cancer, maximum, upper quartile, median, lower quartile, and minimum values, respectively, are 2.317, 0.732, 0.316, 0.12 and 0 for *CHRM1* and 10.027, 4.072, 2.528, 1.094, and 0.02 for *CHRM3*. (**B**) M_1_R and M_3_R protein expression is increased in aberrant crypt foci (ACF). M_1_R and M_3_R immunostaining in ACF and adjacent normal colon. Both M_1_R and M_3_R expression was significantly increased in ACF compared to an adjacent normal colon (*p* < 0.0001 and *p* < 0.001, respectively, respectively; n = 6 per group). Bars represent means ± SE. au, arbitrary units. (**C**) Representative images of M_1_R and M_3_R immunostaining in ACF in tissues from the resected colons of three patients with familial adenomatous polyposis; ACF are delineated by red arrows. Yellow arrows indicate non-dysplastic glands. Size bars for M_1_R and M_3_R staining images respectively: Patient 1, 300 and 500 µm; Patient 2, 500 and 300 µm; Patient 3, 300 and 200 µm. (**D**) M_1_R and M_3_R protein expression is increased in colon adenomas. Both M_1_R and M_3_R expression was significantly increased in adenomas compared to an adjacent normal colon (*p* = 0.0344 and *p* < 0.0001, respectively; n = 12–24 per group). Bars represent means ± SE. au, arbitrary units. (**E**) Representative images of M_1_R and M_3_R immunostaining in colon adenomas. Dysplastic glands are delineated by red arrows. Yellow arrows indicate non-dysplastic glands. Size bars for M_1_R and M_3_R staining images respectively: Patient 1, both 200 µm; Patient 2, both 200 µm; Patient 3, 200 and 300 µm. (**F**) M_1_R and M_3_R protein expression in colon adenocarcinomas. Both M_1_R and M_3_R expression was significantly increased in adenocarcinoma compared to an adjacent normal colon (*p* = 0.02 and *p* < 0.0001, respectively; n = 8 per group). Bars represent means ± SE. au, arbitrary units. (**G**) Representative images of M_1_R and M_3_R immunostaining in colon adenocarcinomas. Size bars for M_1_R and M_3_R staining images respectively: Patient 1, both 400 µm; Patient 2, both 400 µm; Patient 3, both 200 µm. (**H**) M_1_R protein expression in colon cancer lymph node metastases. M_1_R expression was significantly increased in lymph node metastases compared to a normal colon but to a lesser extent than in the primary cancer (*p* = 0.01 and *p* = 0.046, respectively; n = 5 per group). Bars represent means ± SE. au, arbitrary units. (**I**) Representative images of M_1_R immunostaining in a normal colon, colon adenocarcinoma, and lymph node metastases. Size bars as noted on each image. (**J**) M_1_R protein expression in colon cancer liver metastases. M_1_R expression was significantly increased in liver metastases compared to a normal colon to the same degree as in the primary cancer (*p* < 0.001 and *p* < 0.001 compared to a normal colon, respectively; n = 12 per group). Bars represent means ± SE. au, arbitrary units. (**K**) Representative images of M_1_R immunostaining in a normal colon, primary colon adenocarcinomas, and liver metastases. Size bars as noted on each image. Scoring of M_1_R and M_3_R immunostaining for all tissues, including normal colon tissues, was quantified using an analog scale (0, no staining; 4, maximal staining) by a senior pathologist masked to the antibody used. In panels B, D, F, H, and J, solid black circles represent individual tissue scores.

Based on relatively small datasets, *CHRM3*, the gene encoding M_3_R, was reported to be overexpressed in 60–80% of colon cancers [3,4]. However, little was reported regarding *CHRM1* expression in colon cancer or relative *CHRM1* versus *CHRM3* expression. To fill these gaps in knowledge, we performed an in-silico analysis of a publicly available online database, UALCAN, which contains larger colon cancer datasets [9]. This analysis confirmed increased *CHRM3* mRNA levels in colon cancer compared to adjacent normal colon; median *CHRM3* transcript levels were elevated in 286 adenocarcinomas compared to 41 normal colon samples [2.528 vs. 1.272 transcripts per million, respectively (*p* = 0.002)] (Figure 1A). In contrast, *CHRM1* transcript levels in the same dataset were modestly, but significantly (*p* = 0.044), reduced in colon cancer compared to a normal colon (Figure 1A). Analysis of the UALCAN dataset [9] revealed that *CHRM1* transcript levels were approximately 30% lower in colon cancer compared to a normal colon [0.316 versus 1.03 transcripts per million, respectively (*p* = 0.044)] (Figure 1A). Moreover, *CHRM1* transcript levels in colon cancer were significantly lower than *CHRM3* transcript levels (*p* < 0.0005; Figure 1A). We recently confirmed these findings using a smaller dataset in which colon cancer tissue samples were matched to adjacent normal tissue samples [4]. Collectively, these findings support the finding of increased *CHRM3* expression but reduced *CHRM1* expression in colon cancer.

### 3.2. Relative M_1_R and M_3_R Protein Expression in Progressive Colon Neoplasia

For a variety of reasons, including post-transcriptional regulation, mRNA expression may not precisely reflect protein expression. It has been estimated that mRNA expression may account for only 40–60% of the variance in protein expression [10,11,12,13,14]. Hence, we sought to confirm that changes in muscarinic receptor protein expression mirrored those we observed in mRNA expression. Yet, measuring muscarinic receptor protein expression is challenging due primarily to the lack of muscarinic receptor subtype-specific antibodies for immunoblotting, a problem hindering the entire field of G protein-coupled receptor research [15]. As an alternative strategy to assess relative M_1_R and M_3_R protein expression, we used subtype-selective antibodies for immunohistochemistry (IHC), an approach that also allowed us to compare receptor expression in normal and neoplastic areas within the same specimen. As recommended by Jositsch et al. to address the lack of subtype specificity of many anti-muscarinic receptor antibodies [16], we used anti-M_1_R and -M_3_R antibodies from Alomone Labs that were used previously for IHC by our lab [3] and others [17], and whose specificity we had validated using intestinal tissues harvested from wild-type, M_1_R- and M_3_R-deficient, and dual M_1_R/M_3_R-deficient mice.

#### 3.2.1. Relative M_1_R and M_3_R Protein Expression in Aberrant Crypt Foci

We used IHC to perform a comprehensive examination of the relative expression of M_1_R and M_3_R along the continuum from normal colonic mucosa to invasive cancer. Initially, we examined M_1_R and M_3_R staining in aberrant crypt foci (ACF), the first histological manifestation of colon neoplasia [18,19,20,21]. To obtain sufficient ACFs to achieve meaningful comparisons, we examined archived deidentified colon tissues from six individuals with familial adenomatous polyposis (FAP). In FAP, *APC* gene mutations result in aberrant β-catenin signaling and exuberant colon neoplasia (i.e., abundant ACF), thereby facilitating the acquisition and immunohistochemical comparison of M_1_R and M_3_R expression. Staining intensity in Figure 1 was quantified using an analog scale [0 (no staining) to 4 (maximal staining)] by a senior pathologist masked to the antibody used and tissue source. As shown in Figure 1B, compared to adjacent normal colon, both M_1_R and M_3_R expression levels were increased approximately three-fold in ACF compared to adjacent normal colon (*p* < 0.001 and *p* < 0.01 for M_1_R and M_3_R, respectively). As shown by representative images (Figure 1C), even within ACF, the visual intensity of M_1_R and M_3_R staining progressively increased commensurate with advancing degrees of dysplasia. In both normal colon and ACF, we detected no significant difference in the levels of M_1_R compared to M_3_R staining intensity (Figure 1B). Based on these findings, we concluded that M_1_R and M_3_R expression levels were similar in normal colon epithelium and in the earliest stages of colon neoplasia.

#### 3.2.2. Relative M_1_R and M_3_R Protein Expression in Colon Adenomas

Next, we compared M_1_R and M_3_R staining levels in a normal colon to more advanced colon neoplasia, tubular adenomas. As shown in Figure 1D, both M_1_R and M_3_R staining intensity, as scored on an analog scale by a senior pathologist masked to tissue source, was greater in adenomas compared to a normal colon (*p* = 0.0344 and *p* < 0.0001). In contrast to our findings in ACF, the intensity of M_3_R staining in adenomas was significantly greater than that for M_1_R staining (*p* < 0.0001; Figure 1D,E). Unlike our findings with ACF (Figure 1C), for both adenomas and the adjacent normal colon, M_3_R staining intensity exceeded that for M_1_R staining intensity (Figure 1E). We believe there are at least two likely explanations for these differences. First, unlike sporadic colon neoplasia represented by the samples tested in Figure 1E, aberrant β-catenin signaling in all epithelial cells in patients with FAP drives increased expression of both M_1_R and M_3_R in ‘normal’ epithelial cells. Second, increased M_3_R staining in a normal colon adjacent to the adenomas shown in Figure 1E is likely due to a ‘field effect’, wherein a field of chromosomal instability surrounds the area of neoplasia [22]. Representative images shown in Figure 1E highlight the differences between M_1_R and M_3_R expression in normal and adenomatous colon crypts in tissues from the same patients.

#### 3.2.3. Relative M_1_R and M_3_R Protein Expression in Primary Colon Adenocarcinomas

Next, in tissue samples from eight individuals with colon cancer, we compared M_1_R and M_3_R staining in a normal colon and adjacent adenocarcinomas. In this set of samples, M_3_R and M_1_R staining intensity was the same in a normal colon (Figure 1F). As shown in Figure 1F, in adenocarcinomas, staining intensity for both M_1_R and M_3_R was greater than that in a normal colon (*p* = 0.02 and *p* < 0.0001). Nonetheless, the intensity of M_3_R staining in adenocarcinomas was again significantly greater than that for M_1_R staining (*p* = 0.03; Figure 1F). Representative images shown in Figure 1G highlight differences between M_1_R and M_3_R staining intensity in colon cancer samples obtained from the same individuals.

#### 3.2.4. M_1_R Protein Expression in Colon Cancer Lymph Node and Liver Metastases

Previously, we reported that although M_3_R expression was increased in primary colon cancers compared to an adjacent normal colon, M_3_R expression was only minimally elevated in lymph node and liver metastases from the same patients; M_3_R expression in metastases was not significantly increased compared to a normal colon [3]. To determine if the same was true of M_1_R expression, we compared relative M_1_R expression in samples of a normal colon, primary colon cancer, and lymph node and liver metastases from the same patients (Figure 1H–K). In contrast to our findings with M_3_R expression [3], we were surprised to find that M_1_R expression was significantly greater in lymph node and liver metastases compared to a normal colon (*p* = 0.046 and *p* < 0.001, respectively, Figure 1H,J) and M_1_R expression in metastases was not significantly different than that in the respective primary tumors (Figure 1H–K).

Collectively, the findings depicted in Figure 1 allowed us to reach several conclusions. First, while *CHRM1* gene expression was modestly but significantly reduced in colon cancer compared to normal tissue (Figure 1A), M_1_R protein expression levels were significantly increased in both colon adenomas (Figure 1D) and adenocarcinomas (Figure 1F) compared to a normal colon. Second, M_3_R protein expression was significantly greater than M_1_R expression in both adenomas (Figure 1D) and adenocarcinomas (Figure 1F); in both adenomas and adenocarcinomas, M_3_R staining intensity was approximately two-fold greater than that for M_1_R. Third, in contrast to our findings for M_3_R expression, compared to expression levels in primary colon cancers, M_1_R expression remained increased in lymph node and liver metastases (Figure 1H,J).

Collectively, these findings indicate several points of divergence between M_1_R and M_3_R expression in progressive colon neoplasia, from ACF to adenomas, primary colon cancers, and colon cancer metastases. To gain additional mechanistic insights into the role of these muscarinic receptor subtypes in colon neoplasia, we explored the relative effects of M_1_R and M_3_R activation in human colon cancer cells.

### 3.3. Relative Expression of CHRM1 and CHRM3 in Human Colon Cancer Cells

**Figure 2:**
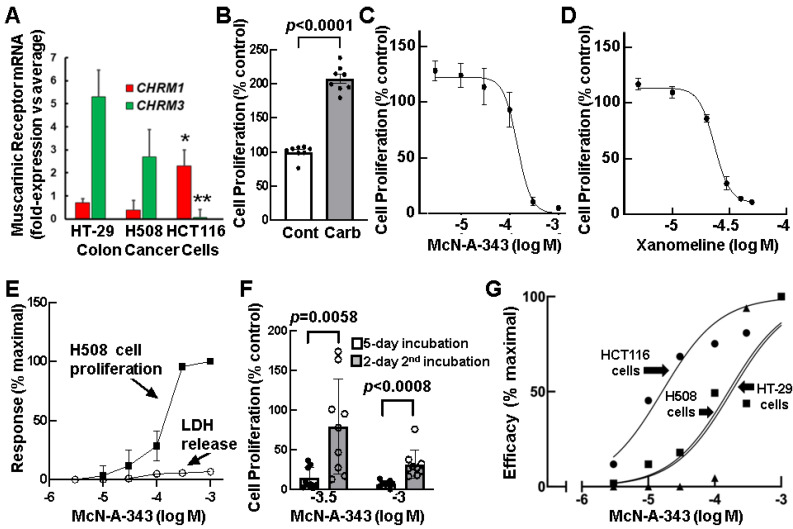
Selective activation of M_1_R dose-dependently and reversibly attenuates human colon cancer cell proliferation. (**A**) Relative expression of *CHRM1* and *CHRM3* mRNA in three established human colon cancer cell lines. *, ** *p* < 0.05 and 0.01 vs. HT-29 cells. Gene expression was normalized to *β*_2_-*microglobulin* (*B2M*) and qPCR data were analyzed using the comparative C_T_ (2^−ΔΔCT^) method. n = 8 for each cell line. (**B**) Effect of treating H508 cells with a non-selective muscarinic agonist. Cells were incubated with or without the test agent for five days before proliferation was measured as described in Methods. Cont, control; Carb, carbachol. Solid black circles represent values for individual replicates. (**C**) Effect of increasing concentrations of McN-A-343 on H508 cell proliferation. Cells were incubated with or without increasing concentrations of McN-A-343 for five days before proliferation was measured as described in Methods. n = 4. (**D**) Effect of increasing concentrations of xanomeline on H508 cell proliferation. Cells were incubated with or without increasing concentrations of xanomeline for five days before proliferation was measured as described in Methods. n = 4. (**E**) Comparison of McN-A-343-induced changes in H508 cell proliferation and LDH release. H508 cells were incubated with or without increasing concentrations of McN-A-343 for five days before proliferation and LDH release into the media was measured as described in Methods. LDH, lactate dehydrogenase. n = 4. (**F**) McN-A-343-induced attenuation of colon cancer cell proliferation is reversible. H508 cells were incubated with 0.3 and 1 mM McN-A-343 for five days (open bars). Cells were then washed and re-incubated for an additional 2 days in media without McN-A-343 (shaded bars). Cell proliferation after the initial incubation with McN-A-343 and following the additional two-day incubation without McN-A-343 was measured as described in Methods. Symbols represent values for individual replicates. (**G**) Comparison of maximal responses observed with increasing concentrations of McN-A-343 in H508 (■), HT-29 (▲), and HCT116 (●) human colon cancer cells. Cells were incubated with or without increasing concentrations of McN-A-343 for five days and cell proliferation was measured as described in Methods. The dose–response curves shown were created using the nonlinear regression feature in GraphPad Prism (GraphPad Prism version 9.5.1, Boston, MA, USA). n = 3–4; for visual clarity, error bars are not shown. n, number of replicates.

To explore the relative expression of M_1_R and M_3_R in established human colon cancer cells, we selected three commonly used cell lines to examine the relative expression of *CHRM1* and *CHRM3* mRNA (Figure 2A); HT-29, H508, and HCT116 cells were used in many of our in vitro studies of colon cancer cell function [23,24]. Whereas these cell lines are well-characterized (all have mutated *APC* and/or *CTNNB1*) [25], their relative expression of *CHRM1* and *CHRM3* was not previously examined. Using qPCR, in HT-29 and H508 cells, we detected substantially higher relative expression of *CHRM3* compared to *CHRM1* mRNA (Figure 2A); H508 and HT29 cells express three- and five-fold, respectively, more *CHRM3* than *CHRM1*. In contrast, HCT116 cells, expressed higher relative levels of *CHRM1* mRNA, thus providing us with experimental colon cancer cell lines with a broad range of relative expression of *CHRM1* and *CHRM3* for our studies (Figure 2A).

### 3.4. Effects of Selective M_1_R Activation on Human Colon Cancer Cell Proliferation

To explore the role of the M_1_R muscarinic receptor subtype in regulating colon cancer cell proliferation, we employed previously validated orthosteric modulators of M_1_R activation. First, we tested the effects of McN-A-343, a M_1_R selective agonist developed over 60 years ago [26], on well-characterized H508 human colon cancer cells that express M_3_R at relatively higher levels than M_1_R (M_1_R^low^; Figure 2A) and whose rates of proliferation respond robustly to treatment with non-subtype-selective muscarinic agonists like acetylcholine and carbamylcholine (carbachol) [23]. Although McN-A-343 may interact with other muscarinic receptor subtypes (e.g., M_4_R), in tissues with limited expression of M_4_R, its use can be helpful to distinguish actions resulting from M_1_R agonism [27,28,29]. Whereas 100 µM carbachol stimulated a two-fold increase in H508 cell proliferation (Figure 2B), treating cells with 10 µM to 1 mM McN-A-343 progressively reduced H508 cell proliferation (Figure 2C). Then, to confirm that M_1_R agonism inhibited colon cancer cell proliferation, we tested the effects of another putative M_1_R selective agonist, xanomeline [30,31,32]. As we observed with McN-A-343, increasing concentrations of xanomeline progressively reduced H508 cell proliferation (Figure 2D). In H508 cells, xanomeline was an approximately six-fold more potent inhibitor of colon cancer cell proliferation than McN-A-343 (IC_50_ xanomeline vs. McN-A-343 = 23.4 µM vs. 136.8 µM).

We used two approaches to exclude non-specific actions of these agents on colon cancer cell viability. First, to determine if cellular toxicity could account for the effects of McN-A-343 on cell proliferation, we measured the cellular release of lactate dehydrogenase (LDH), a commonly used indicator of cell membrane damage that is stable for long periods under our in vitro test conditions [33]. As shown in Figure 2E, even at the highest McN-A-343 concentration used (1 mM), we observed minimal LDH leakage from cells; Triton X-100 was used as a positive control. These results suggested that activating M_1_R in human colon cancer cells selectively stimulates anti-proliferative cell signaling, not cell damage. Next, to test the reversibility of the effects of 0.3 and 1 mM McN-A-343 on cell proliferation, we incubated H508 cells with the M_1_R agonist for two days and then washed and re-incubated cells in culture medium without McN-A-343 for an additional three days. As shown in Figure 2F, compared to cells that had been incubated with McN-A-343 for a full five days, cells re-incubated in the absence of the M_1_R agonist resumed proliferation, findings consistent with effects mediated by reversible M_1_R activation. These findings also supported the conclusion that the effects of the selective M_1_R agonist were not a consequence of cell toxicity.

We next performed experiments to explore the possibilities that stimulation of cellular senescence or apoptosis might explain the results observed with McN-A-343. To test for changes in cell senescence, we determined if treating H508 cells with McN-A-343 altered senescence associated-β-galactosidase staining, a validated marker of premature senescence [34]. As shown by representative images in Supplementary Figure S1A, treatment with McN-A-343 did not induce changes in β-galactosidase staining. To test for McN-A-343-induced apoptosis, we measured changes in total and cleaved caspase-3 expression [35,36]. Treating H508 cells with the highest concentrations of McN-A-343 resulted in a modest reduction in total caspase-3 expression. Nonetheless, as demonstrated by the representative gel shown in Supplementary Figure S1B, treating H508 cells with either a non-selective (carbachol) or selective M_1_R (McN-A-343) agonist did not alter cleaved caspase-3 levels. Based on these findings, we concluded that neither induction of cellular senescence nor apoptosis explained the anti-proliferative effects we observed in cells treated with selective M_1_R agonists.

To confirm that the effects of the M_1_R agonists were not idiosyncratic to H508 cells and to explore the effects of varying relative expression levels of M_1_R and M_3_R (Figure 1A), we examined the effects of increasing concentrations of McN-A-343 on HT-29 (M_1_R^low^) and HCT116 (M_1_R^high^) cells. As shown in Figure 2G, we observed similar inhibitory actions of McN-A-434 on cell proliferation when we tested increasing concentrations of the M_1_R agonist in H508 and HT-29 human colon cancer cells; both cell lines are M_1_R low (Figure 2A). However, the dose–response for McN-A-434-induced inhibition of colon cancer cell proliferation was shifted substantially to the left in HCT116 cells compared to both H508 and HT-29 cells; the half-maximal effective concentration (EC_50_) was 7.89 µM for HCT116 cells, 136.8 µM for H508 cells, and 548.1 µM for HT-29 cells (Figure 2G). We believe the nearly two orders of magnitude increase in potency for the M_1_R agonist in HCT116 cells compared to HT-29 cells is most likely due to the substantially higher relative level of M_1_R expression in M_1_R^high^ HCT116 cells (Figure 2A) [37,38,39].

**Figure 3:**
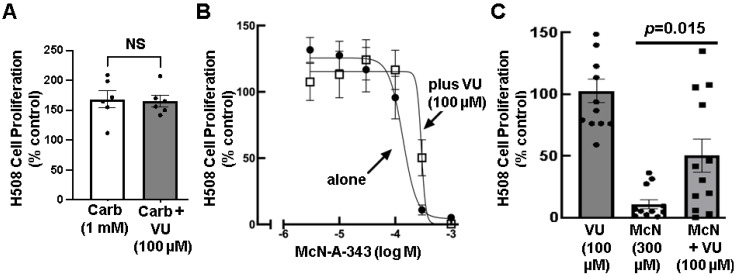
Selectively inhibiting M_1_R activation attenuates the effects of M_1_R-selective but not non-selective muscarinic receptor agonists. (**A**) Selective inhibition of M_1_R activation does not alter carbachol-induced cell proliferation. H508 colon cancer cells were pre-incubated for 30 min with 100 µM VU0255035 before adding 1 mM carbachol and incubating cells for an additional 5 days. Cell proliferation was measured as described in Methods. Each symbol represents the result of a separate experiment. n = 6. (**B**) Selective inhibition of M_1_R activation attenuates the effects of McN-A-343 on cell proliferation. H508 colon cancer cells were pre-incubated for 30 min with 100 µM VU0255035 before adding the indicated concentrations of McN-A-343 and incubating cells for an additional 5 days. Cell proliferation was measured as described in Methods. n = 4. (**C**) Pre-incubating H508 cells for 30 min with 100 µM VU0255035 before adding 300 µM McN-A-343 and incubating cells for an additional 5 days significantly diminished the ability of the selective M_1_R agonist to inhibit cell proliferation. Cell proliferation was measured as described in Methods. n = 12. n, number of replicates, shown by the symbols.

Next, we determined if blocking M_1_R agonism by pre-treating cells with a selective M_1_R inhibitor would mitigate the effects of treating cells with McN-A-343. First, we tested the effects of VU0255035 (VU), a selective M_1_R antagonist [40], on carbachol-induced H508 cell proliferation. As shown in Figure 3A, preincubating cells with a maximal concentration of VU did not alter carbachol-induced cell proliferation, supporting the observation that carbachol is an M_3_R-predominant agonist. Then, we tested the effect of pretreating cells with a fixed concentration of VU on the dose–response curve for McN-A-343-induced inhibition of colon cancer cell proliferation. As shown in Figure 3B, pre-incubation with VU shifted the McN-A-343 dose–response curve significantly to the right (IC_50_ with McN-A-343 alone was 136.8 µM versus 293.0 µM for McN plus VU; *p* = 0.0165). Moreover, as shown in Figure 3C, preincubating cells with 100 µM VU to block the effects of 300 µM McN-A-343 significantly preserved cell proliferation. The highest concentration of VU tested, 100 µM, did not by itself alter basal colon cancer cell proliferation (Figure 3C). However, whereas cell proliferation with McN-A-343 alone was reduced by 90% of control, preincubating cells with VU, attenuated the effects of McN-A-343 such that cell proliferation was reduced by only 50% of control (*p* = 0.015). Collectively, these findings supported the concept that activating M_1_R inhibits colon cancer cell proliferation.

### 3.5. Inhibiting EGFR and p38 MAPK Activation Attenuates the Inhibitory Actions of an M_1_R Agonist on Colon Cancer Cell Proliferation

**Figure 4:**
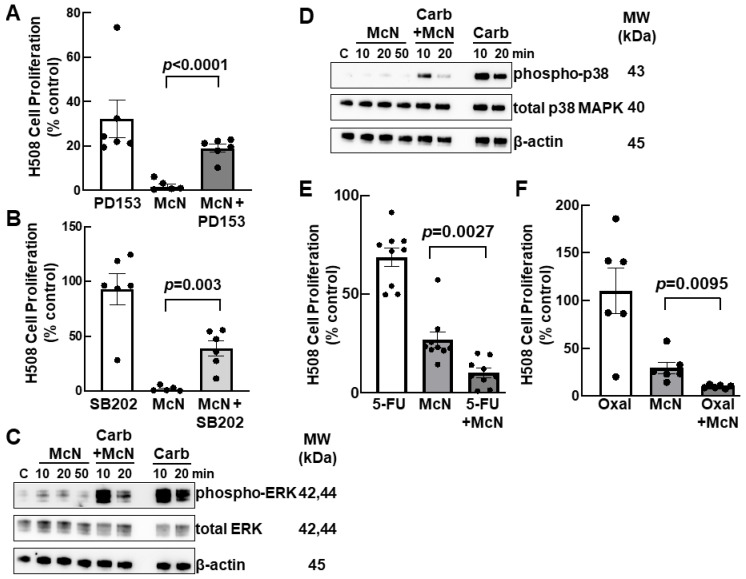
Inhibiting EGFR and p38 MAPK activation attenuates and combining chemotherapeutic agents with an M_1_R agonist augments inhibition of colon cancer cell proliferation. (**A**) Inhibiting EGFR activation attenuates basal colon cancer cell proliferation and partially reverses the actions of a M_1_R agonist. H508 cells were treated for 5 days with 5 µM PD153035 (PD153), 300 µM McN-A-343 (McN), or the combination of PD153035 plus McN-A-343. (**B**) Inhibiting p38 MAPK activation partially reverses the actions of a M_1_R agonist on cell proliferation. H508 cells were treated for 5 days with 10 µM SB202190 (SB202), 300 µM McN-A-343 (McN), or the combination of SB202190 plus McN-A-343. For (**A**,**B**), cell proliferation was measured after a 5-day incubation as described in Methods; symbols represent the results of separate incubations. (**C**) Pre-incubating colon cancer cells with McN-A-343 attenuates carbachol-induced ERK activation. H508 cells were incubated with the vehicle (**C**), 1 mM McN-A-343 (McN), and 1 mM carbachol (Carb) for the times indicated. For the combination of McN-A-343 plus carbachol, cells were pre-incubated with 1 mM McN-A-343 for 30 min before adding carbachol for an additional 10- or 20-min incubation. Membranes were probed with antibodies against total and phosphorylated ERK. (**D**) Pre-incubating colon cancer cells with McN-A-343 attenuates carbachol-induced p38 MAPK activation. H508 cells were incubated with the the vehicle (control; **C**), 1 mM McN-A-343 (McN), and 1 mM carbachol (Carb) alone for the times indicated. For the combination of McN-A-343 plus carbachol, cells were pre-incubated with 1 mM McN-A-343 for 30 min before adding carbachol for an additional 10- or 20-min incubation. Membranes were probed with antibodies against total and phosphorylated p38 MAPK. For (**C**,**D**), β-actin was used as a loading control, and the immunoblots shown are representative of three separate experiments. (**E**) Combining a M_1_R agonist with 5-FU augments the inhibition of colon cancer cell proliferation. H508 cells were incubated for 5 days with 100 µM 5-FU, 200 µM McN-A-343, or the combination of 5-FU plus McN-A343 (McN). n = 9 (**F**) Combining a M_1_R agonist with oxaliplatin augments the inhibition of colon cancer cell proliferation. H508 cells were incubated for 5 days with 10 µM oxaliplatin (oxal), 200 µM McN-A-343, or the combination of 5-FU plus McN-A343 (McN). n = 6. For (**E**,**F**), cell proliferation was measured after a 5-day incubation as described in Methods; symbols represent the results of separate incubations. The whole blot of Figure 4C,D is Figure S3. Symbols in Figure 4A,B,E,F represent values for individual replicates.

To identify relevant signaling proteins downstream of M_1_R activation, we used selective inhibitors for several kinases whose actions were previously implicated in post-muscarinic receptor signaling in colon cancer; we had previously validated the actions of these inhibitors in human colon cancer cells [23]. First, we determined whether maximal concentrations of any of these kinase inhibitors altered McN-A-343-induced inhibition of cell proliferation. As shown in Supplementary Figure S2, treating H508 cells with inhibitors of Src, phosphoinositide 3-kinase (PI3K), mitogen-activated protein kinase kinase (MEK), and protein kinase C (PKC) did not alter the actions of McN-A-343. In contrast, pretreating H508 cells with 5 µM PD153035, a selective inhibitor of epidermal growth factor receptors (EGFR), or 10 µM SB202190, a selective inhibitor of p38 mitogen-activated protein kinase (MAPK), partially reversed the inhibitory actions of McN-A-343 on cell proliferation (Figure 4). As shown in Figure 4A, treating H508 cells with the EGFR inhibitor reversed the effects of McN-A-343 by approximately 50% (*p* < 0.0001). As shown in Figure 4B, treating H508 cells with a p38 MAPK inhibitor had an effect of similar magnitude, reducing McN-A-343-induced effects by approximately 50% (*p* = 0.003). We previously showed that transactivation of EGFR occurs downstream of muscarinic receptor activation [24].

As both ERK and p38 MAPK activation are downstream of EGFR activation [30], we pursued these potential roles for EGFR and p38 MAPK by examining the effects of McN-A-343 on ERK and p38 MAPK activation (i.e., phosphorylation). As post-receptor kinase activation occurs quickly after muscarinic receptor activation [23,24], we examined both ERK and p38 MAPK phosphorylation at early time points, 10 to 50 min. As shown in Figure 4C and Figure 4D, respectively, treating cells with 1 mM McN-A-343 alone did not alter ERK or p38 MAPK phosphorylation during this time span. In contrast, treating cells with 1 mM carbachol stimulated robust ERK and p38 MAPK phosphorylation at 10 min which was diminished by 20 min (Figure 4C,D). Notably, the combination of McN-A-343 plus carbachol attenuated both ERK and p38 MAPK phosphorylation at 20 min; carbachol-induced activation of p38 MAPK was attenuated after both 10 and 20 min of incubation (Figure 4D). None of these treatments altered the expression of total ERK or total p38 MAPK (Figure 4C,D). Collectively, these findings support the conclusion that EGFR, ERK, and p38 MAPK activation play a role in McN-A-343-mediated inhibition of colon cancer cell proliferation.

### 3.6. Combining an M_1_R Agonist with Conventional Colon Cancer Chemotherapy Enhances Inhibition of Cell Proliferation

Lastly, we were curious to see if combining an M_1_R agonist with currently used agents for colon cancer chemotherapy had therapeutic potential. Interestingly, we found that treating H508 human colon cancer cells with a submaximal dose of McN-A-343 (200 µM) in combination with either 100 µM 5-fluorouracil (5-FU) (Figure 4E) or 10 µM oxaliplatin (Figure 4F) potentiated the anti-proliferative effects of both chemotherapeutic agents acting alone. These findings suggest that adding a M_1_R agonist to conventional chemotherapy for colon cancer may have therapeutic benefit.

## 4. Discussion

The work presented here expands our understanding of the divergent roles M_1_ and M_3_ muscarinic receptors play in colon cancer progression, and the findings that they appear to be reciprocal regulators of colon cancer cell proliferation opens the door to therapeutic opportunities. *CHRM3* and M_3_ muscarinic receptors are commonly over-expressed in colon cancer [3] and genetic ablation of *Chrm3* or pharmacological inhibition of M_3_R activation in murine models of colon cancer attenuates tumor formation [5]. In contrast, we found that *CHRM1* mRNA expression is significantly reduced in human colon neoplasia [4] and *Chrm1* ablation in mouse models negates the anti-neoplastic effects of *Chrm3* ablation [5]; that is, *Chrm1*/M_1_R expression appears to protect against colon cancer. This concept was again supported in the present work by our interrogation of a large public colon cancer database which confirmed that while *CHRM3* levels were significantly elevated in cancer compared to those in normal colon tissue, *CHRM1* levels were modestly, but significantly, reduced in cancer.

Due to epigenetic and other effects, the functional component of gene expression, protein expression, may not mirror changes in mRNA levels [41,42,43]. To address this concern, we applied previously validated muscarinic receptor-subtype-specific antibodies to examine and compare M_1_R and M_3_R protein expression in progressive stages of colon neoplasia. Notably, we observed that the relative expression of M_3_R compared to M_1_R increases with the progressive pre-metastatic development of colon cancer from ACF to adenomas to primary adenocarcinomas. Whereas M_1_R and M_3_R expression was similar in a normal colon and the expression of both receptor subtypes increased equally in ACF, the ratio of M_3_R to M_1_R expression was significantly enhanced in adenomas and primary colon cancers. It is possible, although admittedly highly speculative, that increased M_1_R expression in colon ACF, adenomas, and cancers is a compensatory mechanism in reponse to greatly increased M_3_R expression and, thus, an anti-neoplastic effort on the part of cells to maintain homeostasis.

It is noteworthy that M_1_R levels in lymph node and liver metastases, although increased compared to normal colon tissue, were not increased compared to primary cancers (Figure 1H–K). We previously reported that M_3_R levels were lower in colon cancer metastases than in primary colon cancers; in fact, levels in metastases were not significantly different compared to levels in normal colon tissue [3]. We believe these findings have several important implications. First, they suggest that colon cancer cells that have already metastasized no longer require the attributes, like enhanced migration and invasion, that accrue from increased M_3_R activation. Second, persistent over-expression of M_1_R in metastatic cells compared to a normal colon suggests that M_1_R is accessible for targeting to attenuate the proliferation of metastatic colon cancer cells, thereby potentially blocking the expansion and, more intriguingly, promoting the regression of colon cancer metastases. Although these suggestions also remain speculative until supported by additional experimental findings, the latter is of key importance to the translational value of our findings. Whereas primary colon cancers rarely result in death as they are readily amenable to endoscopic or surgical resection, metastatic colon cancer almost always becomes resistant to therapy, resulting in a fatal outcome. Hence, a therapeutic approach that may selectively target metastatic colon cancer cells is attractive.

Regarding the translational potential of our findings, we present several lines of evidence to support the concept that M_1_R activation impedes colon cancer cell proliferation. We tested two different selective M_1_R agonists, McN-A-343 and xanomeline, on H508 human colon cancer cells, a cell line previously used in many colon cancer studies. In contrast to carbamylcholine, which robustly stimulated H508 cell proliferation, both M_1_R agonists dose-dependently reduced tumor cell proliferation, effects attenuated by pre-incubating cells with a selective M_1_R inhibitor. Key findings were reproduced in two additional commonly used human colon cell lines, HT-29 and HCT116 cells. Notably, in HCT116 cells, the McN-A-343 inhibition dose–response curve was left-shifted by two orders of magnitude, likely reflecting the observation that this cell line overexpresses M_1_R relative to M_3_R (Figure 2A).

Several experiments provided evidence that the inhibitory effects of M_1_R agonism on cell proliferation were not mediated by test agent toxicity or the induction of cellular senescence or apoptosis; after applying test agents (carbachol and McN-A-343) to colon cancer cells, we detected no meaningful changes in LDH release, senescence-activated β-galactosidase activity, or cleaved caspase-3 generation. Nonetheless, we did observe a modest reduction in total caspase-3 levels in cells treated with the highest McN-A-343 concentrations. Although speculative, it is possible that McN-A-343 reduces expression of caspase-3 by a currently unclear mechanism. Moreover, our findings contrasted with those from investigators who observed that in HEK293 cells overexpressing M_1_R, treatment with carbachol induced apoptosis by an ERK-independent mechanism [44]. We ascribe these divergent outcomes to experimental conditions, including the use of different cell types, i.e., HEK293 versus human colon cancer cells. Perhaps the most important difference is that in contrast to our work which utilized established human tissue-derived cell lines with naturally occurring levels of M_1_R expression, carbachol-induced cell death was observed in HEK293 cells expressing supra-physiological levels of M_1_R, a caveat highlighted by the authors [44]. Indeed, our findings are reassuring in that they speak to the likely safety of exposing normal tissues to M_1_R agonists and support the hypothesis that the anti-proliferative effects of M_1_R agonists derive from specific alterations in post-M_1_R signaling. This hypothesis was supported by the results of cell proliferation experiments that examined the effects of pre-treating cells with selective kinase inhibitors before adding an M_1_R agonist and detected M_1_R agonist-induced changes in kinase activation by immunoblotting for phosphorylated (activated) kinases.

To identify potential post-receptor mediators of M_1_R agonist-induced attenuation of colon cancer cell proliferation, we examined the effects of blocking key kinases in the muscarinic receptor signal transduction pathway [23]. This set of experiments revealed that activation of a signaling cascade involving EGFR, ERK, and p38 MAPK likely plays a role in mediating M_1_R agonist-induced attenuation of cell proliferation; validated EGFR and p38 MAPK inhibitors significantly attenuated the actions of McN-A-343 on cell proliferation (Figure 4A,B) and McN-A-343 treatment attenuated carbachol-induced ERK and p38 MAPK phosphorylation (activation) (Figure 4C,D). We cannot presently explain why inhibition of MEK, a kinase upstream of ERK activation did not alter McN-A-343-induced attenuation of cell proliferation (Supplementary Figure S2B); this finding suggests the possible presence of an alternative route of ERK activation downstream of M_1_R activation. Control experiments using selective inhibitors of other relevant kinases clearly demonstrated their lack of involvement in the effects of McN-A-343 (Supplementary Figure S2).

Our work has limitations. These include the use of selective but not necessarily specific muscarinic receptor agonists and inhibitors. Although all the agents we used were validated in previous studies, we must acknowledge the possibility of off-target effects on other muscarinic receptor subtypes, e.g., M_4_R. We also acknowledge the possibility that field effects in ‘normal’ tissue adjacent to colon cancer cells may impact the analysis of muscarinic receptor expression depicted in Figure 1; that is, muscarinic receptor expression may be even lower in normal tissue more distant from the site of cancer. Moreover, whereas we detected limited variability in M_1_R and M_3_R expression in tissue from a normal colon, there was extensive variability in receptor expression in neoplastic tissues (Figure 1). Admittedly, more mechanistic investigation is required to fully understand the signaling pathways underlying the actions of M_1_R activation. Nonetheless, these experiments clearly implicate the EGFR, ERK, p38 MAPK signaling cascade in mediating the anti-proliferative effects of M_1_R activation. Future experiments will seek a deeper mechanistic understanding of precisely how M_1_Rs regulate cell proliferation, if this requires proximity between M_1_R and M_3_R as was reported for other plasma membrane G protein-coupled receptors with reciprocal relationships [45], and whether a similar reciprocal relationship exists between M_1_R and M_3_R activation in other gastrointestinal cancers—current evidence suggests this is highly likely. Lastly, animal studies are required to demonstrate both the efficacy and safety of M_1_R agonism in models of both sporadic and colitis-associated colon cancer; a report that McN-A-343 treatment reduces inflammation and oxidative stress in a murine model of ulcerative colitis suggests promise for the latter indication [46].

In summary, we have demonstrated a novel anti-proliferative effect of M_1_R activation in human colon cancer cells that may explain why *CHRM1*/M_1_R levels in colon cancer are relatively reduced compared to *CHRM3*/M_3_R levels. The findings reported here are likely to have clinical importance. For example, our analysis of M_1_R versus M_3_R expression in progressive colon neoplasia (Figure 1) suggests the potential of using the M_3_R:M_1_R expression ratio as a biomarker to predict which cancers are better suited for treatment with M_3_R inhibitors versus M_1_R agonists (i.e., M_1_R^low^ vs. M_1_R^high^). Additionally, our experiments examining the effects of combining an M_1_R agonist with conventional colon cancer chemotherapeutics (Figure 4E,F) suggest that M_1_R agonism, alone or in combination with these agents, is a potential therapeutic strategy worthy of further investigation.

## 5. Conclusions

Novel insights into the structural biology and mechanisms whereby muscarinic receptors regulate fundamental functions of the central and enteric nervous systems are driving drug development [1]. There is focused interest within the pharmaceutical industry to develop selective M_1_R agonists to treat a host of neurological and psychiatric disorders, including schizophrenia and Alzheimer disease [47,48]. For example, a xanomeline-trospium formulation is currently in clinical trials. To reduce peripheral cholinergic side-effects, this agent combines, xanomeline, an M_1_R-selective blood–brain barrier permeable agonist, with trospium, a non-selective muscarinic receptor inhibitor that does not cross the blood–brain barrier. The trospium moiety is intended to mitigate the off-target peripheral cholinergic side-effects of xanomeline, e.g., blurred vision, excess sweating and salivation, and urinary frequency. Marketed as KarXT by Karuna Therapeutics, this agent demonstrated an acceptable safety profile in Phase 2 clinical trials and is currently in Phase 3 trials for psychosis and schizophrenia [49,50]. Similar agents could be designed or repurposed to treat cancers of the colon and other GI organs in which muscarinic receptor signaling plays an important role. Based on our collective findings, designing drugs that exhibit dual selective M_3_R antagonism and M_1_R agonism may have great therapeutic promise to treat advanced colon cancer.

## Data Availability

The data presented in this study are available on request from the corresponding author.

## Supplementary Materials

The following supporting information can be downloaded at: https://www.mdpi.com/article/10.3390/cancers15194766/s1, Figure S1: Markers of senescence and apoptosis are not altered in M_1_R agonist-treated human colon cancer cells; Figure S2: Inhibitors of protein kinase C-α (PKCα), mitogen activated kinase kinase (MEK), phosphoinositide 3-kinase, and Src do not alter the actions of an M_1_R agonist on human colon cancer cell proliferation; Figure S3: Original uncut gels for immunoblots. Table S1: Primers for human genes used in this study; Original uncut gels for immunoblots.
