# Supplementary material for: Selective Activation of M1 Muscarinic Receptors Attenuates Human Colon Cancer Cell Proliferation
Source: Cancers (Basel). 2023 Sep 28;15(19):4766. doi: 10.3390/cancers15194766 (PMC10571583; doi:10.3390/cancers15194766)

Supplementary Materials - Figures, Table, and Uncut Gels

Supplementary Figures:

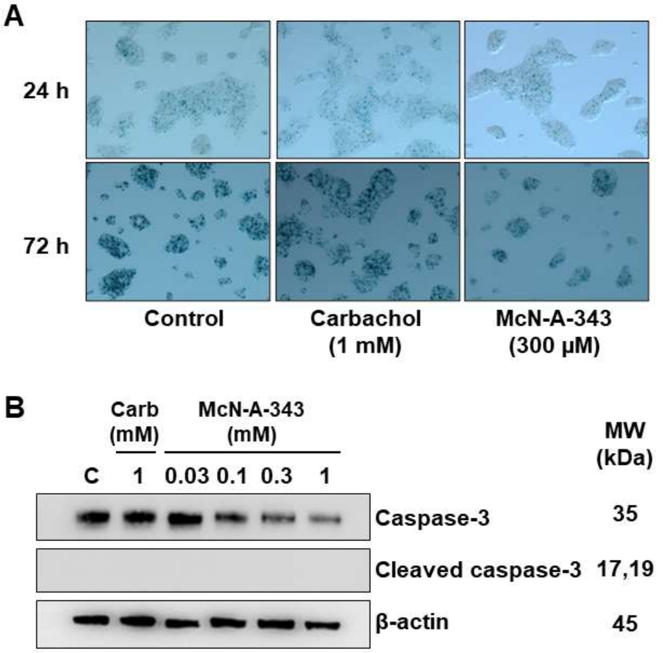

Supplementary Figure S1. Markers of senescence and apoptosis are not altered in M1R agonist-treated human colon cancer cells. (**A**) Non-selective and M1R-selective agonists do not alter senescence-activated β-galactosidase activity in H508 colon cancer cells. H508 cells were treated with test agents for 24 h and 72 h before staining for senescence-activated β-galactosidase. Representative images are shown. (**B**) Non-selective and M1R-selective agonists do not alter cleaved caspase-3 levels in H508 colon cancer cells. H508 cells were stimulated for 48 h with 1 mM carbachol (Carb) and increasing concentrations of McN-A-343. Following incubation, cell lysates were probed with antibodies against total caspase-3, cleaved caspase-3, and β-actin (loading control). A representative gel is shown.

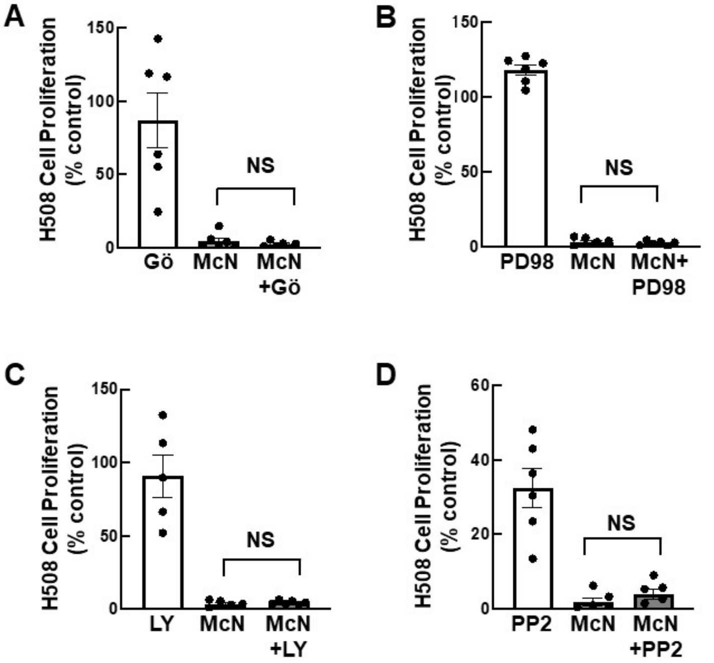

Supplementary Figure S2. Inhibitors of protein kinase C-α (PKCα), mitogen activated kinase kinase (MEK), phosphoinositide 3-kinase, and Src do not alter the actions of an M1R agonist on human colon cancer cell proliferation. (**A**) Inhibiting PKCα activation does not alter the actions of a M1R agonist. H508 cells were treated for 5 days with 5 μM Gö6983 (Gö), 300 μM McN-A-343 (McN), or the combination of Gö6983 plus McN-A-343. (**B**) Inhibiting MEK activation does not alter the actions of a M1R agonist. H508 cells were treated for 5 days with 10 μM PD98059 (PD98), 300 μM McN-A-343 (McN), or the combination of PD98059 plus McN-A-343. (**C**) Inhibiting phosphoinositide 3-kinase activation does not alter the actions of a M1R agonist. H508 cells were treated for 5 days with 10 μM LY294002 (LY), 300 μM McN-A-343 (McN), or the combination of LY294002 plus McN-A-343. (**D**) Inhibiting Src activation does not alter the actions of a M1R agonist. H508 cells were treated for 5 days with 10 μM PP2, 300 μM McN-A-343 (McN), or the combination of PP2 plus McN-A-343. For (**A**), (**B**), (**C**), and (**D**), cell proliferation was measured after a 5-day incubation as described in Methods; symbols represent the results of separate incubations. NS, not significant.

Supplementary Table:

Supplementary Table S1. Primers for human genes used in this study.

Genes	Forward primer (5′-3′)	Reverse primer (5′-3′)
h_*CHRM1*	GGGCAGTGCTACATCCAGTTCC	CGTGCTCGGTTCTCTGTCTCCC
h_*CHRM3*	TGGTTTGATGCTCCTACCTG	AGGACAGAGGAGTGGACCAG
h_*B2M*	GAGGCTATCCAGCGTACTCC	ATGGATGAAACCCAGACACA

B2M, beta-2 microglobulin

Figure S3. Original uncut gels for immunoblots: The following are original immunoblot images visualized and exported through the chemiluminescence channel from the ChemiDoc Touch Imaging System. Red boxes delineate cropped versions shown in manuscript figures. In red, we also indicated the proteins targeted for analysis. Each lane is labeled in black with respective conditions and loading order.

Figure 4c

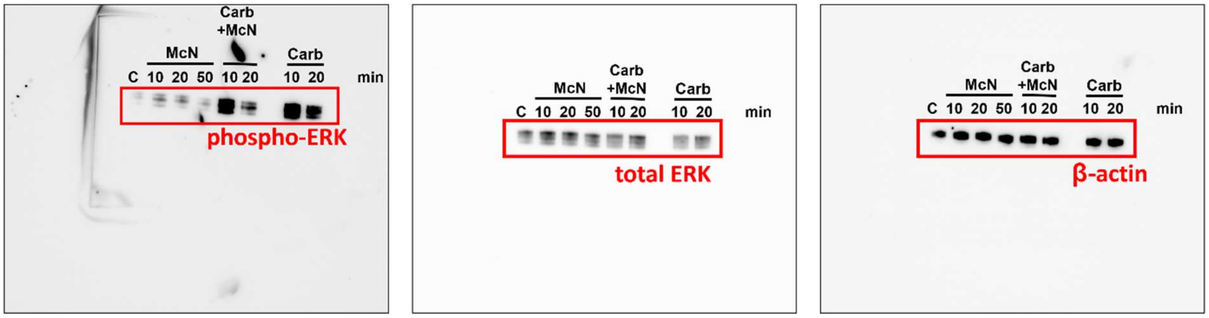

Figure 4d

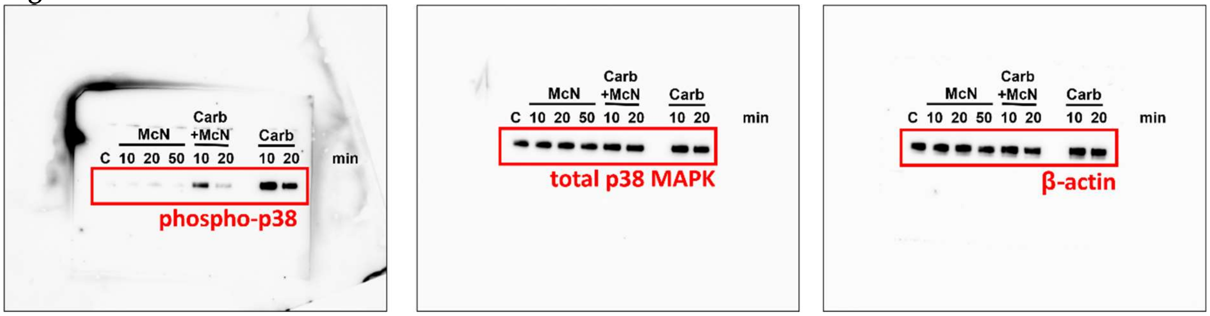

Supplementary Figure S1B

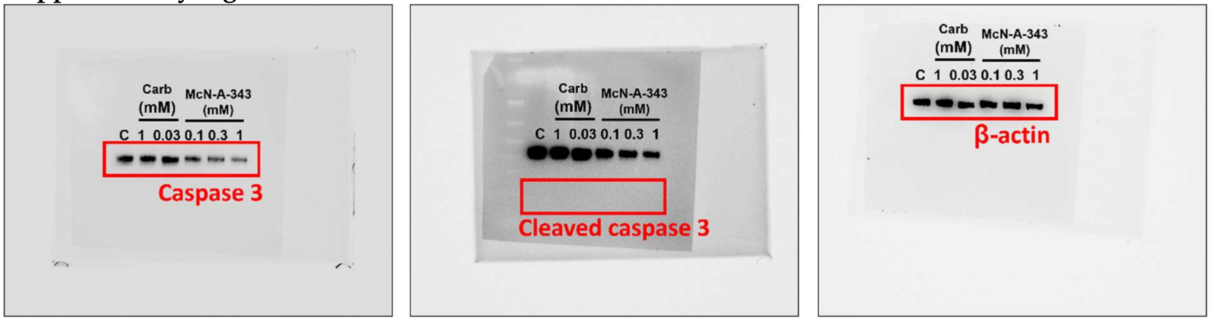

Supplement: Supplementary file 1 [file cancers-15-04766-s001.zip › cancers-2601850-supplementary.pdf]
